# Understanding diabetes in patients with HIV/AIDS

**DOI:** 10.1186/1758-5996-3-2

**Published:** 2011-01-14

**Authors:** Sanjay Kalra, Bharti Kalra, Navneet Agrawal, AG Unnikrishnan

**Affiliations:** 1 Dept of Endocrinology, Bharti Hospital, Karnal, India; 2 Dept of Medicine, DOTC, Gwalior, India; 3 Dept of Endocrinology, AIMS, Kochi, India

## Abstract

This paper reviews the incidence, pathogenetic mechanisms and management strategies of diabetes mellitus in patients with human immunodeficiency virus (HIV) and acquired immunodeficiency syndrome (AIDS). It classifies patients based on the aetiopathogenetic mechanisms, and proposes rational methods of management of the condition, based on aetiopathogenesis and concomitant pharmacotherapy.

## Introduction

Patients with human immunodeficiency virus (HIV) and acquired immunodeficiency syndrome (AIDS) are increasing in number, partly due to improved screening, earlier diagnosis, better methods of treatment, and greater accessibility to, as well as acceptance of therapy. Figures from the United Nations reveal that the total number of patients with HIV is 33 million, with 2.7 million new infections in 2007 [1].

Improved methods of detection of HIV, earlier diagnosis, and better management have helped in improving the survival of these patients. The availability of, and access to, potent retroviral and anti-infective therapy has translated into lesser acute morbidity and mortality, and thus, a longer lifespan. This is turn, has meant an increase in the chronic complications of HIV encountered in clinical practice [2]^.^

HIV/AIDS patients, therefore, frequently present with diabetes and metabolic complaints. As treatment of HIV develops, and access to therapy improves, the incidence of HIV-associated diabetes is bound to grow. An international cross-sectional study of 788 HIV-infected adults recruited at 32 centers has studied the metabolic syndrome prevalence using International Diabetes Federation (IDF) and U.S. National Cholesterol Education Program Adult Treatment Panel III (ATPIII) criteria, relative to body composition (whole-body dual-energy X-ray absorptiometry and abdominal computed tomography), lipids, glycemic parameters, insulin resistance, leptin, adiponectin, and C-reactive protein (CRP) [3]^.^

The prevalence of metabolic syndrome was 14% (*n *= 114; 83 men) by IDF criteria and 18% (*n *= 139; 118 men) by ATPIII criteria. Half of the patients (49%) exhibited two or more features of metabolic syndrome but were not classified as having the syndrome because they had normal or low waist circumferences or waist-to-hip ratios. Metabolic syndrome was more common in those currently receiving protease inhibitors (*P *= 0.04). Type 2 diabetes prevalence was five- to ninefold higher in those with metabolic syndrome [3].

In another study, the incidence of new-onset diabetes in HIV-infected persons was significantly high. Over 130,151 person-years of follow-up (PYFU), in the Data Collection on Adverse Events of Anti-HIV Drugs (D:A:D) Study, diabetes was diagnosed in 744 patients (incidence rate of 5.72 per 1,000 PYFU [95% CI 5.31-6.13]). The incidence of diabetes increased with cumulative exposure to combination ART. The strongest relationship with diabetes was exposure to stavudine, while treatment with zidovudine and didanosine was also associated with an increased risk of diabetes [4].

Management of HIV/AIDS is gradually expanding to include the chronic, and metabolic, complications of the disease, and the adverse effects associated with its treatments. The aim of this article is to review the management strategies for diabetes in patients in HIV/AIDS, while understanding the pathophysiologic mechanisms predisposing to their condition.

## Diabetes and HIV: Classification

Three subgroups of patients with diabetes and HIV can be identified: Patients with preexisting diabetes who contract HIV, those who are diagnosed to have diabetes at onset of HIV infection, and others who develop hyperglycemia after start of therapy. These subgroups need to be managed differently, as the mechanisms of metabolic dysregulation vary in them.

Patients with HIV will certainly have the same rates of diabetes as seen in the background population. Because of their relatively younger age, HIV patients may have lower incidence of preexisting diabetes when they get infected. However, as they grow older, they may develop diabetes in the normal course of events. Certain metabolic factors related to HIV, and to HIV therapy, may increase the incidence of diabetes among them.

These factors will be discussed in the following paragraphs. This knowledge is of importance to treating physicians, as it will help them plan their mode of treatment.

## Aetiopathogenesis

With a high prevalence of diabetes in the background population, it stands to reason that the same predisposing factors will operate in patients with HIV.

Apart from this, however, HIV patients present with metabolic syndrome, altered glucose metabolism, dyslipidemia and lipodystrophy.

Many risk factors contribute to the development of metabolic syndrome in such patients. These include advancing age, male gender, longer duration of HIV infection, low CD4 count, high viral burden, high body mass index, greater waist circumference or waist- to- hip ratio, lower socio economic class, and certain ethnic backgrounds or culture [5,6]

Impaired glucose tolerance, and insulin resistance are noted to precede weight loss in patients with HIV [5-9]. Insulin resistance, rather than insulin deficiency, is usually implicated in the pathogenesis of diabetes in HIV-infected patients. According to earlier reports, evidence of islet cell autoimmunity, or beta cell destruction has not been seen in HIV patients[10].

Autoimmune diabetes, however, has recently been reported to develop in some HIV-infected patients after immune restoration during HAART. Three Japanese patients presenting with diabetes after receipt of HAART have been shown to develop antibodies to glutamic acid decarboxylase, at a time when CD4 counts shot up suddenly. The postulate is that recovery of immune function predisposes to autoimmune disease, in the form of type 1 diabetes (T1DM) [11].

The type of diabetes associated with HIV may be classified as type 2 diabetes (T2DM), rather than T1DM, in the vast majority of patients..

Concurrent use of opiates, however, may alter beta cell function[12], while heroin addiction is associated with insulin resistance. No specific mechanisms of action have been proposed for these effects. No studies have been reported on alpha-cell function in people with concurrent HIV and diabetes.

HIV infection is linked with hepatitis C infection (HCV), which is associated with insulin resistance and diabetes, due to increased intrahepatic tumour necrosis factor (TNF α) and hepatic steatosis[10]. These factors increase the risk of diabetes in a patient suffering from concurrent HIV and HCV infection. Persons with HCV who are 40 years of age or older are greater than 3 times more likely to have diabetes than those of the same age without HCV infection[13]. In a Korean study of 1,117 patients with chronic viral hepatitis, 21% of HCV-infected patients had a diagnosis of diabetes, compared with 12% of patients infected with hepatitis B virus [14]

HIV is also associated with various endocrine abnormalities, including those of the growth hormone axis. These include deficiency of growth hormone, as well as growth hormone resistance. Growth hormone deficiency may contribute to insulin resistance in HIV-infected patients [15].

The increased accumulation of visceral fat, with wasting of subcutaneous fat, noted in these patients, creates higher levels of inflammatory cytokines such as TNF α. This in turn leads to diabetes or impaired glucose tolerance by increasing insulin resistance [16].

HIV-infected subjects with metabolic syndrome show disturbances in inflammation and adipokines: they have higher CRP (5.5 ± 7.0 vs. 3.9 ± 6.0 mg/l, *P *< 0.003) and leptin (9 ± 9 vs. 4 ± 6 ng/ml, *P *< 0.0001) and lower adiponectin (12 ± 8 vs. 15 ± 10 μg/ml, *P *< 0.0001) levels. This may contribute to the pathogenesis of diabetes[3].

Viral factors which contribute to diabetes risk are an increase in viral burden of 0.5 log over a 6 month period, a lower CD4 count, and longer duration of HIV infection [5]. In general, people with severe, long-standing HIV infection are more prone to developing diabetes.

The above listed factors partly explain the pathogenesis of diabetes detected during the natural history of HIV infection.

The major contributor to hyperglycemia in HIV/AIDS, however, is iatrogenic. The past few decades have seen remarkable improvement in the clinical outcome of HIV patients, thanks to highly active antiretroviral therapy (HAART). Benefits of HAART include suppression of viral load, improvement in CD4 count, decrease in opportunistic infections and length of hospital stay, and reduction in mortality [17,18].

HAART however, has also lead to an increase in metabolic dysfunction, including insulin resistance, diabetes dyslipidemia and lipodystrophy [12].

This constellation of abnormalities is also called antiretroviral-associated diabetes, and is consistent with the clinical picture of T2DM, rather than T1DM [10].

A recent analysis has found that diabetes is four fold more common is HIV-infected men exposed to highly active anti retroviral therapy (HAART) than in HIV seronegative men [19]^. ^HAART is based on the use of a class of drugs known as protease inhibitors (PIs), which have been used extensively as antiretroviral agents. The various PIs used include atazanavir, darunavir, saquinavir and ritonavir.

PIs have been shown to increase insulin resistance and reduce insulin secretion, by interfering with GLUT-4 mediated glucose transport. Risk factors for development of diabetes with PI therapy include positive family history of diabetes, weight gain, lipodystrophy, old age and hepatitis C infection [20]. PIs interfere with cellular retinoic acid-binding protein type 1 (CRABP 1) that interacts with peroxisomal proliferator-activated receptor (PPAR) γ. Inhibition of PPAR-γ promotes adipocyte inflammation, release of free fatty acids and insulin resistance [21]. Hyperglycemia resolves in almost all patients when PIs are discontinued [21].

All PIs do not have the same metabolic effects. Indinavir induces insulin resistance with no effect on lipid metabolism, whereas lopinavir and ritonavir increase fasting triglycerides and free fatty acids, but do not worsen insulin sensitivity. Indinavir and retonavir both block GLUT -4, but no such effect is noted with amprenavir, and atazanzvir. HIV-infected patients treated for 12 weeks with nelfinavir, indinavir, liponavir or saquinavir, demonstrate alterations in first phase insulin release with a 25% reduction in β-cell dysfunction [20,21].

This implies that there is no class effect of PIs on diabetes, and that various PIs should be studied individually with respect to their metabolic effects [21]. The therapeutic implication is that treating physicians should be aware of the predominant mechanism of diabetes linked to each drug, so that appropriate anti-diabetic therapy can be chosen. For example, a patient with diabetes due to nelfinavir, indinavir, liponavir or saquinavir therapy will benefit more from insulin than from insulin sensitizer therapy.

The other class of drugs which is used is the nucleoside analogs (reverse transcriptase inhibitors) (NRTIs). It was earlier felt that NRTIs were less likely to cause metabolic abnormalities. A recent study, which analyzed 130 151 person years of exposure, however, has shown that these drugs, too, increase the risk of diabetes [4].

The risk is highest with stavudine, but is also significant with zidovudine and didanosine. Proposed mechanisms include insulin resistance, lipodystrophy, and mitochondrial dysfunction [22]. These mechanisms may be evident only in HIV-infected persons treated for long periods of time with NRTIs [22]. This does not mean that HAART should not be prescribed to patients with HIV and diabetes. One should be aware of the adverse metabolic effects of these drugs, and take proactive steps to prevent and manage these.

It is postulated that PIs confer acute metabolic risks, while NRTIs confer cumulative risks of diabetes in predisposed, exposed persons. Exposure to a combination of NRTI and indinavir (a PI) has been shown to be an additional risk factor for onset of diabetes [22].

Anti-retroviral drugs are not the only iatrogenic culprits in HIV-associated diabetes. Drugs used to manage comorbid conditions associated with AIDS may also cause diabetes. Pentamidine, which is used to prevent and treat P. carinii associated pneumonia, can cause β-cell toxicity, with acute hypoglycemia followed by later diabetes. Factors associated with increased risk of hypoglycemia are longer and higher dosage of pentamidine, as well as renal insufficiency. The group of patients which progressed to diabetes had low C peptide levels, suggestive of β- cell destruction [23,24]^. ^This mechanism of action implies that insulin will be the only rational choice of treatment in patients with pentamidine-induced diabetes. About 8% of all pentamidine-treated patients will develop hypoglycemia [25] This is of importance because P. carinii pneumonia is the most common index infection at time of diagnosis of AIDS [26].

Megesterol acetate, which is used as an appetite stimulant, predisposes to diabetes because of its intrinsic glucocorticoid like activity, increased caloric intake and weight gain [27]. Hypoglycemia has been noted to resolve once megesterol is stopped, and to recur on rechallenging.

Patients on HAART may also be predisposed to diabetes because of the improved nutritional status and weight gain that accompanies effective treatment of HIV.

## Screening for Diabetes

Patients with HIV should be screened for diabetes at diagnosis, at onset of HAART therapy, and three to six months after HAART. While certain professional bodies advise fasting blood glucose as a screening tool [28], the predominant role of insulin resistance in the development of the illness implies that postprandial glucose values, or an oral glucose tolerance test, should also be performed as part of screening procedures.

One may follow guidelines for the general population, and screen all HIV patients at diagnosis and at regular intervals, with both fasting and postprandial glucose values. Venous samples should be taken for diagnosis, rather than fingerprick values. Glucose challenge tests may be indicated in select cases. A1c has not been recommended as a diagnostic test in HIV/AIDS.

## Strategies to Manage Hyperglycemia

Guidelines for management of diabetes in the OPD set up have been discussed in detail elsewhere. However, these do not focus on HIV-associated diabetes.

Management strategies for diabetes are somewhat different in HIV patients than in the general population. The unique features of diabetes management in HIV are listed and discussed below:

### 1. General Measures

Modification of risk factors such as hypertension, dyslipidemia, and platelet function should be done through non-pharmacological and pharmacological methods, as in non-HIV infected patients. Precipitating factors such as tuberculosis should be searched for, and treated aggressively. Concurrent sexually transmitted diseases, pruritus vulvae, and balanoposthitis should be screened for, and managed appropriately. A complete physical examination of the genitalia is mandatory. Untreated genito-urinary infection is a common cause of poorly controlled hyperglycemia.

Dyslipidemia is common in HIV, and treatment guidelines are available to help practitioners handle this complication [28]. Pravastatin and fluvastatin are safe to use with ritonavir. Atorvastatin and rosuvastatin should be used with caution, while simvastatin is contraindicated in patients on this PI [29]. This is because of the common cytochrome P450 isoenzyme. pathways used for metabolism

Hypertension, too, is a frequent comorbid condition. The routinely used antihypertensives, such as angiotensin converting enzyme (ACE) inhibitors and angiotensin receptor blockers (ARBs) may not be optimal choices in patients with HIV [29]. Captopril has been associated with Kaposi's sarcoma, hepatotoxicity and neurotoxicity. Enalapril is linked with muscle pain, weakness and diarrhoea. ARBs may compete with other drugs that are metabolized by the cytochrome P450 isoenzyme.

### 2. Life Style Modification

Diet, physical activity/exercise and cessation of smoking are as important in HIV-infected as in non infected persons [30,31].

Weekly counseling by a dietician with main emphasis on healthy eating, and limited emphasis on weight loss, has been shown to reduce blood pressure, waist circumference and HbA1c significantly [32], along with a reduction in caloric intake and percentage of calories from saturated fat, as well as increased fibre intake. On the other hand, many patients of HIV are cachexic, and need increased caloric intake to improve general health. This caloric intake should be a balanced diet, following the same proportions that non-HIV infected patients are recommended.

Endurance and resistance exercise have been shown to have positive effects on metabolic parameters in HIV infected patients [33]. Similar results, including decreased blood pressure, increased strength and endurance, lower cholesterol and increased insulin sensitivity [30,34] are seen with weight lifting and aerobic/endurance exercise.

Smoking cessation is critical for optimal health. Newer non nicotine replacement therapy should be used in caution in HIV patients. Varenicline and bupropion may interact with drugs metabolized by the cytochrome P450 isoenzyme, e.g, ritonavir, efavirenz and nelfinavir [35].

### 3. Psychosocial Support

While psychosocial support is an integral part of effective diabetes management, it is of utmost importance in patients who have to handle the double stress of diabetes and HIV.

Strategies to support behaviour change need to be individualized, culture- age - and gender - appropriate, negotiable, flexible and dynamic. The WATER approach, coined at Bharti Hospital, Karnal, India, is an effective method of motivational interviewing [36]. WATER stands for Welcome Warmly, Ask and Assess, Tell the Truth, Explain it with Empathy, and Reassure and encourage to Return. These five points encapsulate the basic principles of diabetes/HIV counseling, and are an easy method of teaching health care providers how to deal with patients.

Coping skills training should be provided to all HIV patients, and should focus on enhancing positive coping strategies, confidence and self-esteem. The AEIOU method is a simple and time-efficient way of doing so [37]. AEIOU implies Ask and Assess coping skills; Explain and Eliminate negative coping methods; Identify and Internalize positive methods of coping; Observe on an Ongoing basis for improvements; and Utilize as well as Upgrade ones' skills continuously.

### 4. Oral Anti Diabetic Drugs (OADs)

OADs are frequently used in patients with T2DM. The patient with coexistent HIV infection, however, poses a special challenge.

This patient is at greater risk of comorbidity such as hepatitis C, tuberculosis or other opportunistic infections. He or she may have severe insulin resistance. The number of concomitant medications is greater, leading to an increased chance of drug interactions. Because of impaired renal and hepatic function, the risk of adverse events and drug toxicity may be higher. Cachexia and impaired appetite may increase the risk of hypoglycemia. Gastrointestinal infections and dysfunction may worsen tolerability of various oral drugs, and alter their absorption [38,39].

One should therefore choose an OAD regime in patients with diabetes and HIV with great care (Additional file 1).

Metformin is the first line drug of choice in most persons with T2DM, but should be used with caution in HIV.

Though it improves insulin sensitivity, it may not be well tolerated by cachexic patients. Metformin is more likely to cause diarrhea than other drugs [40]

It is contraindicated in renal or hepatic dysfunction, and may lead to metformin-associated lactic acidosis (MALA). It should be avoided in combination with drugs such as stavudine, which also increase the risk of lactic acidosis[40]. Abacavir, lamivudine and tenofovir are the least likely drugs to cause elevation of lactate levels.

HIV patients on metformin should be educated about the symptoms of lactic acidosis, including fatigue, weight loss, nausea, abdominal pain, dyspnea, and arrhythmia. Liver-related symptoms such as tender hepatomegaly, edema, ascites and encephalopathy may occur, but jaundice is uncommon [38].

Metformin should be avoided in patients with comorbid infection such as tuberculosis, weight loss, cachexia, ketonuria and lipoatrophy. Further reductions in subcutaneous fat can occur with this drug [40]. This in turn, may worsen lipodystrophy seen with HIV, and lead to a deterioration in various metabolic parameters.

The thiazolidinediones have a mechanism of action which should make them drugs of choice in HIV. The possibility of a slight increase in subcutaneous fat makes them the preferred drug class in patients with lipodystrophy [28]. However, poor responses to peroxisome proliferator-activated receptor-γ agonists and fibrates have been reported in randomized trials in HIV infected patients with T2DM[41]. The drugs are contraindicated in hepatic dysfunction and heart failure. They may cause edema, increase cardiovascular morbidity, worsen osteoporosis, and decrease hematocrit. These side effects prevent wide usage of these drugs in T2DM, as well as HIV-associated diabetes.

Ritonavir and nelfinavir may reduce rosiglitazone concentrations because of a similar metabolic pathway [38]. Few drug interaction studies have been done between antidiabetic drugs and antiretroviral molecules, but the potential for these, and the frequent administration of polypharmacy for other comorbid conditions, implies that insulin is a safer alternative.

Insulin secretagogues such as repaglinide, and sulphonylureas (glimepiride, gliclazide, glibenclamide) are safe, but may not be effective in the face of severe insulin resistance. However, amongst the OADs, they have a faster onset of action, and may be used in appropriate doses, provided there is no ketonuria. The glinides address the defect in first phase insulin secretion that is seen with certain PIs, and may be an appropriate choice of OADs [38].

The newer class of OADs, the polypeptide protease (IV) inhibitors, ie, saxagliptin, sitagliptin, and vildagliptin, have not been studied in patients with HIV and diabetes. There is a theoretical risk of immunodeficiency and exacerbation of infections [42] with these drugs, however, which prevent them from being used as first line drugs in HIV.

Effects similar to those seen in the general population may be expected with incretin mimetics in HIV-infected patients. Liraglutide has recently been reported to improve various indices of insulin sensitivity, including HOMA-IR, blood pressure and weight, apart from achieving effective hypoglycemic control [43]. These properties may make it worthwhile to study the effect of liraglutide and exenatide in HIV-associated diabetes.

### 5. Insulin

Insulin is the drug of choice for management of diabetes with HIV. Insulin has an anabolic effect, is known to reduce inflammatory markers such as TNF-alpha, does not have any interactions with antiretroviral or other drugs, is not contraindicated with renal or hepatic dysfunction, does not reduce appetite or cause gastrointestinal side effects, can correct both insulin deficiency and resistance when given in appropriate doses, and does not increase the risk of cardiovascular disease[44].

Insulin therapy should be initiated at the outset, using basal bolus regime or premixed insulin. The American Association of Clinical Endocrinologists recommends the use of modern insulins or insulin analogues, as they are more predictable in action, and cause less hypoglycemia. The use of traditional human insulins is discouraged [45].

Insulin requirements are high to begin with, and fall after a few weeks, once glucotoxicity is corrected and infection controlled. Insulin requirements may rise as appetite returns to normal and caloric intake increases. Sick patients should be tested for ketonuria [46]. Rapid acting analogues such as aspart insulin may obviate the need for admission in patients with ketonuria [47], and are useful for critically ill patients as well [48].

An average patient will need 1.0 U/kg/day of insulin initially, divided as 60% bolus and 40% basal insulin. In a few weeks, the requirement will come down to 0.5 U/kg/day, and may be met by two or three equal doses of premixed aspart/lispro [49]. Basal insulin is usually not sufficient alone to meet insulin requirements in severely infected patients with HIV/AIDS (personal observation).

HIV-infected patients should be taught how to dispose of lancets, glucose strips, insulin syringes, pens and needles, to prevent HIV transmission.

### 6. Changes in HAART

PI-based regimes should be avoided in patients at high risk of developing diabetes, e.g., those with a history of gestational diabetes, a positive family history of diabetes, or impaired glucose tolerance on screening. Indinavir should be avoided, and replaced with less toxic drugs.

Structured treatment interruption should be avoided as it increases the risk of death, opportunistic disease [50], and myocardial infarction [51].

Patients should be counselled about the potential risks, discomforts and benefits of HAART, and encouraged to follow a healthy lifestyle while monitoring glycemia regularly.

## Management of Pre-Existing Diabetes

Pre-existing T2DM may continue to be managed, after diagnosis of HIV, with the same drug therapy that was being used prior to detection of HIV. Patients should be informed about the chances of worsening hyperglycemia, and educated about the features of ketosis and lactic acidosis. In case glycemic control deteriorates, insulin should be initiated, rather than increasing dosage or number of OADs.

## Management of Diabetes Detected at Diagnosis

Patients diagnosed to have diabetes and HIV together may be treated according to guidelines for non-infected individuals. The initial drug of choice will remain metformin, unless contraindicated or not tolerated. In such cases, it will be prudent to use insulin or low dose meglitinides as a second line therapy. The baseline A1c will determine the choice of therapy to a large extent

## Management of Diabetes Occurring After Haart

Patients developing diabetes after HAART may be treated with OADs, metformin being the first choice. However, control is usually suboptimal (personal experience). Insulin is a better and safer choice, and may be tapered or reduced once control is achieved.

## Conclusion

The effective management of diabetes in HIV infected patients requires a thorough understanding of pathophysiology and pharmacology. The choice should be based on the aetiopathogenesis of the disease. Patients with pre-existing diabetes should be counseled about a possible deterioration in metabolic function, and the chances of drug interactions between OADs and HAART. Patients who are detected to be diabetic at onset of therapy or later, may benefit from insulin. Insulin is a safe and effective method of treating all these patients, irrespective of type of diabetes.

## Conflict of interests

The authors declare that they have no competing interests.

## Authors' contributions

All authors have contributed equally to the concept of the review, the literature search, and the medical writing. All authors have read and approved the final manuscript.

## Supplementary Material

Additional file 1 Table 1: Choosing an OAD in HIV Click here for file
