# Supplementary material for: Understanding diabetes in patients with HIV/AIDS
Source: Diabetol Metab Syndr. 2011 Jan 14;3:2. doi: 10.1186/1758-5996-3-2 (PMC3025836; doi:10.1186/1758-5996-3-2)
Supplement: Additional file 1 — Table 1: Choosing an OAD in HIV [file 1758-5996-3-2-S1.DOCX]

TABLE 1: USE of ANTI-DIABETIC DRUGS in HIV

| drug | advantages | limitations | recommendation |
| --- | --- | --- | --- |
| Metformin | Economical; first line drug in non-infected persons | Gastrointestinal intolerance; potential for lactic acidosis | Use if well tolerated, and if no contraindications are present. |
| Sulfonylureas | effective | May cause hypoglycemia | Use if metformin is contraindicated/not tolerated |
| Thiazolidinediones | Less risk of hypoglycaemia; may correct lipodystrophy | Edema, cardiac safety, osteoporosis | Avoid use unless other drugs are not tolerated |
| DPP-4 inhibitors | Low risk of hypoglycemia | May theoretically exacerbate infections | Avoid use unless other drugs are not tolerated |
| Alpha-glucosidase inhibitors | Low risk of hypoglycemia | Gastrointestinal intolerance | Use if metformin is contraindicated/not tolerated |
| Incretin mimetics | Low risk of hypoglycemia | Gastrointestinal intolerance; loss of weight | Use if weight loss is required |
| Insulin | Effective, safe; no drug interactions; improves appetite; corrects cachexia | May cause hypoglycemia | May use in all situations, in appropriate dosages |
